# Comparison of models of diffusion in Wilms’ tumours and normal contralateral renal tissue

**DOI:** 10.1007/s10334-020-00862-4

**Published:** 2020-07-02

**Authors:** Harriet J. Rogers, Martijn V. Verhagen, Chris A. Clark, Patrick W. Hales

**Affiliations:** 1 Great Ormond Street Institute of Child Health, University College London, 30 Guilford St, Holborn, London, WC1N 1EH UK; 2 Great Ormond Street Hospital, Great Ormond Street, London, WC1N 3JH UK; 3 Centre of Medical Imaging, Division of Medicine, University College London, 2nd Floor Charles Bell House, 43-45 Foley Street, London, W1W 7TS UK

**Keywords:** Wilms’ tumour, Kidneys, Diffusion

## Abstract

**Objective:**

ADC (Apparent Diffusion Coefficient) derived from Diffusion-Weighted Imaging (DWI) has shown promise as a non-invasive quantitative imaging biomarker in Wilms’ tumours. However, many non-Gaussian models could be applied to DWI. This study aimed to compare the suitability of four diffusion models (mono exponential, IVIM [Intravoxel Incoherent Motion], stretched exponential, and kurtosis) in Wilms’ tumours and the unaffected contralateral kidneys.

**Materials and methods:**

DWI data were retrospectively reviewed **(**110 Wilms’ tumours and 75 normal kidney datasets). The goodness of fit for each model was measured voxel-wise using Akaike Information Criteria (AIC). Mean AIC was calculated for each tumour volume (or contralateral normal kidney tissue). One-way ANOVAs with Greenhouse–Geisser correction and post hoc tests using the Bonferroni correction evaluated significant differences between AIC values; the lowest AIC indicating the optimum model.

**Results:**

IVIM and stretched exponential provided the best fits to the Wilms’ tumour DWI data. IVIM provided the best fit for the normal kidney data. Mono exponential was the least appropriate fitting method for both Wilms’ tumour and normal kidney data.

**Discussion:**

The diffusion weighted signal in Wilms’ tumours and normal kidney tissue does not exhibit a mono-exponential decay and is better described by non-Gaussian models of diffusion.

## Introduction

Wilms' tumour is the most common paediatric renal tumour [1], and in Europe patients are treated with chemotherapy prior to surgery to reduce tumour size [2]. Following a full or partial nephrectomy, histological analysis classifies the tumour as a subtype depending on the predominant cell type [3]. Patients will often have multiple MRI scans to monitor response to treatment, with Diffusion Weighted Imaging (DWI) being frequently acquired.

The Apparent Diffusion Coefficient (ADC) can be derived from DWI by applying a mono-exponential fit (Eq. 1) to the diffusion data.

1 $$\mathrm{S}\left(\mathrm{b}\right)={\mathrm{S}}_{0}{\mathrm{e}}^{-\mathrm{b}.\mathrm{A}\mathrm{D}\mathrm{C}}$$

where S(b) is the signal at a given b value, and S_0_ is the signal with no diffusion weighting.

ADC has shown great promise as a quantitative imaging tool in Wilms’ tumour. For example, ADC has been used to distinguish benign from malignant tumours (a subset of this cohort being Wilms’ tumours) [4], separate neuroblastoma from Wilms’ tumour [5], monitor chemotherapy response [6, 7], identify histological subtypes [7], and assist in identifying necrotic Wilms’ tumour tissue [8].

While ADC is a useful parameter, there are other non-Gaussian models (IVIM [9] [Intravoxel Incoherent Motion], stretched exponential [10], and kurtosis [11]) which can be applied to DWI data to produce a wide range of diffusion metrics. IVIM (Eq. 2) is a bi-exponential model which not only describes water movement within the extra-vascular space but also in the randomly oriented micro capillary network. It produces the parameters D (the diffusion coefficient free from the influence of fast-flowing water in the capillary network, referred to as ‘slow’ diffusion), D* (the diffusion coefficient due to the randomly-orientated motion of water in the blood in the capillary network – ‘fast’ diffusion), and f (the volume fraction associated with the fast-flowing component).

2 $$\mathrm{S}\left(\mathrm{b}\right)={\mathrm{S}}_{0}[\left(1-\mathrm{f}\right){\mathrm{e}}^{(-\mathrm{b}.\mathrm{D})}+\mathrm{f}{\mathrm{e}}^{[-\mathrm{b}.\left(\mathrm{D}+{\mathrm{D}}^{\mathrm{*}}\right)]}]$$

The stretched-exponential model (Eq. 3) describes heterogeneity in diffusion within a single voxel, describing the deviation from a mono-exponential decay. It produces the parameters DDC (the distributed diffusion coefficient) and ⍺ (the stretching parameter to describe the deviation from homogenous diffusion).

3 $$\mathrm{S}\left(\mathrm{b}\right)={\mathrm{S}}_{0}{\mathrm{e}}^{(-\left(\mathrm{b}.{\mathrm{D}\mathrm{D}\mathrm{C})}^{\mathrm{a}}\right)}$$

The kurtosis model (Eq. 4) describes the deviation from the displacement of water molecules following a Gaussian distribution and produces the parameters D_k_ (the diffusion coefficient corrected for the non-Gaussian displacement), and K (the kurtosis).

4 $$\mathrm{S}\left(\mathrm{b}\right)={\mathrm{S}}_{0}{\mathrm{e}}^{-\mathrm{b}.{\mathrm{D}}_{\mathrm{k}}+{\mathrm{b}}^{2}{{\mathrm{D}}_{\mathrm{k}}}^{2}\mathrm{K}/6}$$

These models have the potential to provide supplementary information regarding tissue microstructure. Additionally, they have been shown to provide superior descriptions of diffusion data compared to the mono-exponential model in rectal cancer [12], prostate cancer bone metastases [13], ovarian cancer [14] and in healthy renal tissue [15]. However, there is limited research into applying these models in Wilms’ tumour where they may be useful, due to the highly heterogenous cellular environment of the tumour tissue. Furthermore, it is hypothesised that due to the high levels of perfusion in the kidneys, and that IVIM is designed to account for a perfusion-related component in the diffusion signal, this model model may provide a superior fit to this DWI data [16].

The aim of this research was to determine whether these models (IVIM, stretched exponential and kurtosis) provide superior fits to the diffusion-weighted signal compared to a mono-exponential model, in Wilms’ tumours and the contralateral normal kidney. The goodness of fit was calculated using the Akaike Information criterion (AIC) [17], which penalises models containing more free parameters than are supported by the raw data. Additionally, as a secondary aim, Wilms’ tumours were separated by histological subtype to determine whether certain models favoured certain subtypes.

## Materials and methods

### Study population

Institutional ethical approval was granted and waived the need for consent for this single centre study. A 10-year retrospective review (April 2007–March 2017) of the radiology imaging system at our institution was performed for all abdominal MRI data in children with a proven histological diagnosis of Wilms’ tumour. Inclusion criteria were those with multiple b value DWI data (including a maximum b value of 1000 s/mm^2^), and tumour size covering at least 2 axial slices on DWI. DWI with extreme motion artefacts were also removed. MRI data were collected from Wilms’ tumour patients both pre- and post-chemotherapy. Histological subtypes were confirmed post-surgery for a subset of the tumours. For normal kidney data, the contralateral unaffected kidney was used, except in patients with bilateral disease.

### MRI

All imaging was performed on a 1.5 T Siemens Magnetom Avanto scanner equipped with 40 mT/m gradients. Depending on patient size, one or two body matrix coils were used to obtain full coverage (6 element design, Siemens). Patients were either awake or anaesthetised depending on their age.

Multiple b value DWI was obtained for all patients and was acquired during free breathing. The DWI protocol was as follows: 7 or 8 b values in 3 orthogonal directions (0, 50, 100, 250, 500, 750, 1000 s/mm^2^ or 0, 50, 100, 150, 200, 250, 500, 1000 s/mm^2^) slice thickness: 6 mm, TR/TE: 2800 ms/89 ms, field of view: 350 × 350 mm, voxel size: 1.4 × 1.4  ×  6 mm, number of slices: 19, matrix size: 128 × 96 × 19. Nine averages were acquired for each b value, and trace images (mean over 3 directions) were used for analysis. Standard clinical sequences were also acquired in conjunction, including fat-suppressed T_1_w before and after administration of gadolinium-based contrast; full details of the clinical imaging sequences can be found in [18].

### Post-processing

Diffusion data were processed using the trace images and in-house model fitting routines designed in Matlab (version 2019a, MathWorks Inc., Natick, MA, USA) on a voxel-by-voxel basis using four different models of diffusion: mono exponential (Eq. 1), IVIM (Eq. 2), stretched-exponential (Eq. 3), and kurtosis (Eq. 4).

In each case S_0_ was defined as the signal at b = 0, and for the mono-exponential model a linear fit of ln(S/S_0_) against all b values was performed. For the non-Gaussian models, fitting was performed using the Levenberg–Marquardt nonlinear least squares algorithm (using the ‘lsqcurvefit’ function in Matlab), across all b values (except for the IVIM model). For the IVIM model, firstly, a linear fit of ln(S/S_0_) against b was calculated at high b values (200–1000 s/mm^2^) to determine the value of D. Following this, D* and f were fit simultaneously (with a fixed D). D* had no constraints on upper boundaries, and f was constrained between 0 and 1. For the stretched-exponential model DDC had no upper boundary conditions, and α was constrained between 0 and 1. For kurtosis neither D_k_ nor K were constrained by upper boundaries, and K had a lower bound of 0.

### Regions of interest (ROIs)

**Fig. 1:**
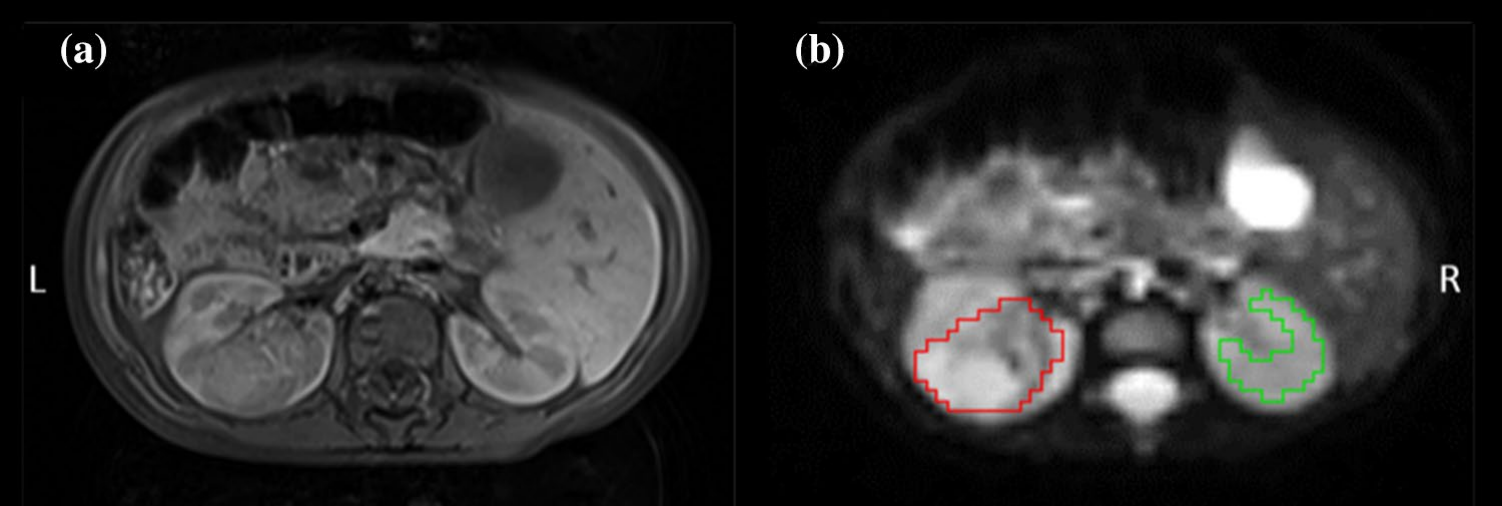
An example of a representative Wilms’ tumour. Displayed is a central axial slice of a T_1_w image **(a)** and b0 image **(b)**. The abdomen is shown at the level of the kidney of a Wilms’ tumour patient post-chemotherapy (age at time of scan: 1.22 years). ROIs are shown surrounding the tumour (red) and normal renal tissue (green)

ROIs were generated using Mango Software (Research Imaging Institute, UTHSCSA). ROIs were drawn on the b0 images around the entire tumour volume, these were edited and verified by a radiologist specialising in paediatric radiology (M.V. 3 years dedicated paediatric radiology). Normal kidney tissue was also defined on the b0 images using the contralateral kidney (excluding those with bilateral disease) around entire kidney volume and areas of high flow, such as the areas which surround the renal pelvis were excluded; an example can be seen in Fig. 1. All analysis regarding model comparisons was confined to these ROIs.

### Model comparison analysis

AIC was used to compare the four models (mono exponential, IVIM, stretched exponential and kurtosis). For every voxel within the tumour ROIs and normal kidney ROIs, AIC was calculated per model. The mean AIC was calculated across the entire ROI volume per model. Mean AIC values for each model were then compared using a one-way repeated measures ANOVA with a Greenhouse–Geisser correction to account for non-equal variance, and post hoc tests were performed using the Bonferroni correction. A significant difference was defined as *p* < 0.05. ANOVAs were calculated for the entire Wilms’ tumour and normal kidney populations, as well as within different subgroups (pre-chemotherapy, post-chemotherapy, and different b value acquisitions [7 and 8 b value ranges]). Additionally, models were compared between Wilms’ tumour histological subtypes to determine whether a certain subtype favoured a particular model. The post-chemotherapy data were used for this comparison as it was the nearest time point to histology.

## Results

### Study population

**Fig. 2:**
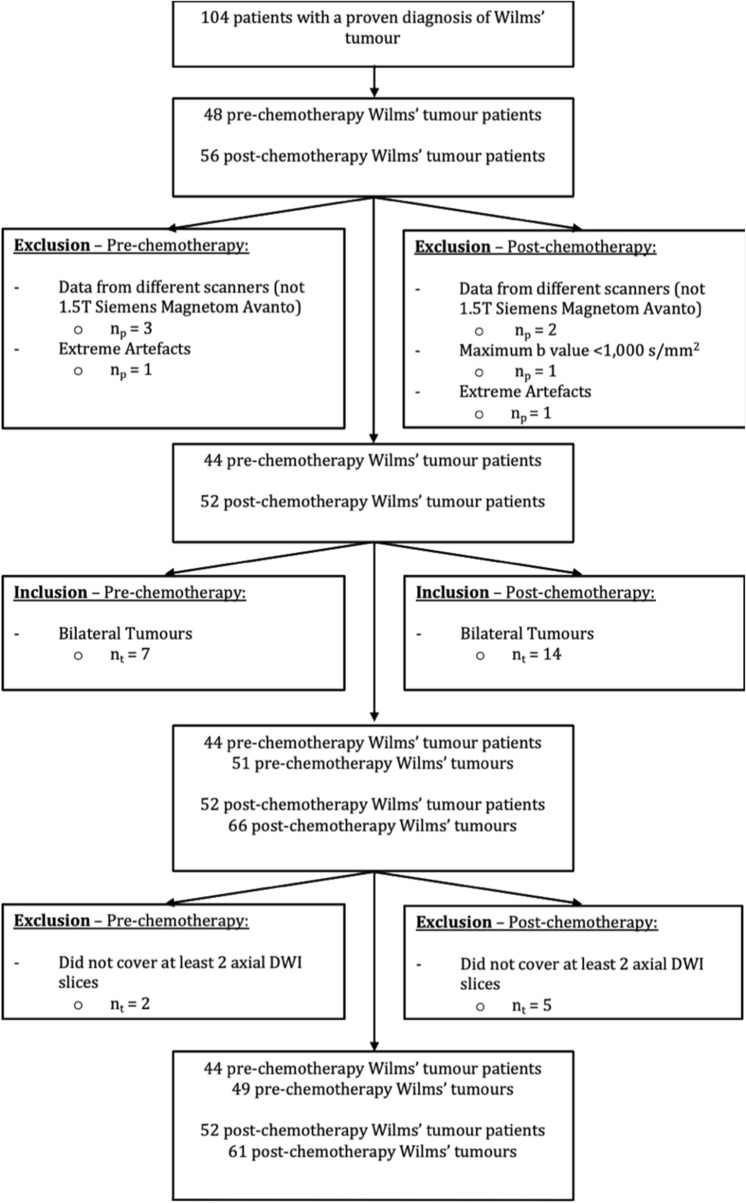
Flowchart of study population showing inclusions and exclusion criteria. *DWI *diffusion-weighted imaging. *n*_*p*_number of patients, *n*_*t*_number of tumours

A total of 110 Wilms’ tumours were included for diffusion model comparison analysis; consisting of 49 pre-chemotherapy and 61 post-chemotherapy tumours (38 of the pre-chemotherapy tumours were included as part of the 61 post-chemotherapy cohort). A flow chart detailing inclusions and exclusions of cases can be seen in Fig. 2. The mean age of patients at their pre-chemotherapy scan was 2.43 years (SD: 2.2), and the mean age at their post-chemotherapy scan was 3.0 years (SD: 2.8).

The diffusion data were acquired using either 7 or 8 b values (0, 50, 100, 250, 500, 750, 1000 s/mm^2^ or 0, 50, 100, 150, 200, 250, 500, 1000 s/mm^2^). This was due to the protocol changing during the period of this study, for reasons un-related to this study. Forty-nine tumours had the 7 b value protocol (22 pre-chemotherapy and 27 post-chemotherapy), and 61 tumours had the 8 b value protocol (27 pre-chemotherapy and 34 post-chemotherapy).

Of the 61 post-chemotherapy tumours, 56 had histologically confirmed subtypes: 7 blastemal, 9 epithelial, 13 stromal, 8 regressive, 18 mixed and 1 completely necrotic. Subtypes were defined according to SIOP-2001 protocol [3].

The contralateral unaffected kidney was used as the normal kidney data. Due to the need to exclude bilateral cases, a total of 75 normal kidney datasets were included; 38 from patients who had received chemotherapy and 37 from patients who had not. Of the 75 normal kidney datasets 31 had the 7 b value protocol (15 pre- chemotherapy and 16 post-chemotherapy), and 44 had the 8 b value protocol (22 pre- and 22 post-chemotherapy).

### Wilms’ tumour results

One-way ANOVAs with a Greenhouse–Geisser correction revealed that AIC values differed significantly between diffusion models, in all conditions: entire cohort: (*F*(1.08, 117.91) = 157.08, *p* = 1.68 × 10^–24^), pre-chemotherapy: (*F*(1.05, 50.53) = 79.35, *p* = 3.11 × 10^–12^), post-chemotherapy: (*F*(1.13, 67.92) = 85.92, *p* = 1.34 × 10^–14^), 7 b values: (*F*(1.21, 58.16) = 76.23, *p* = 2.10 × 10^–13^), and 8 b values: (*F*(1.04, 62.49) = 95.51, *p* = 1.68 × 10^–14^).

**Fig. 3:**
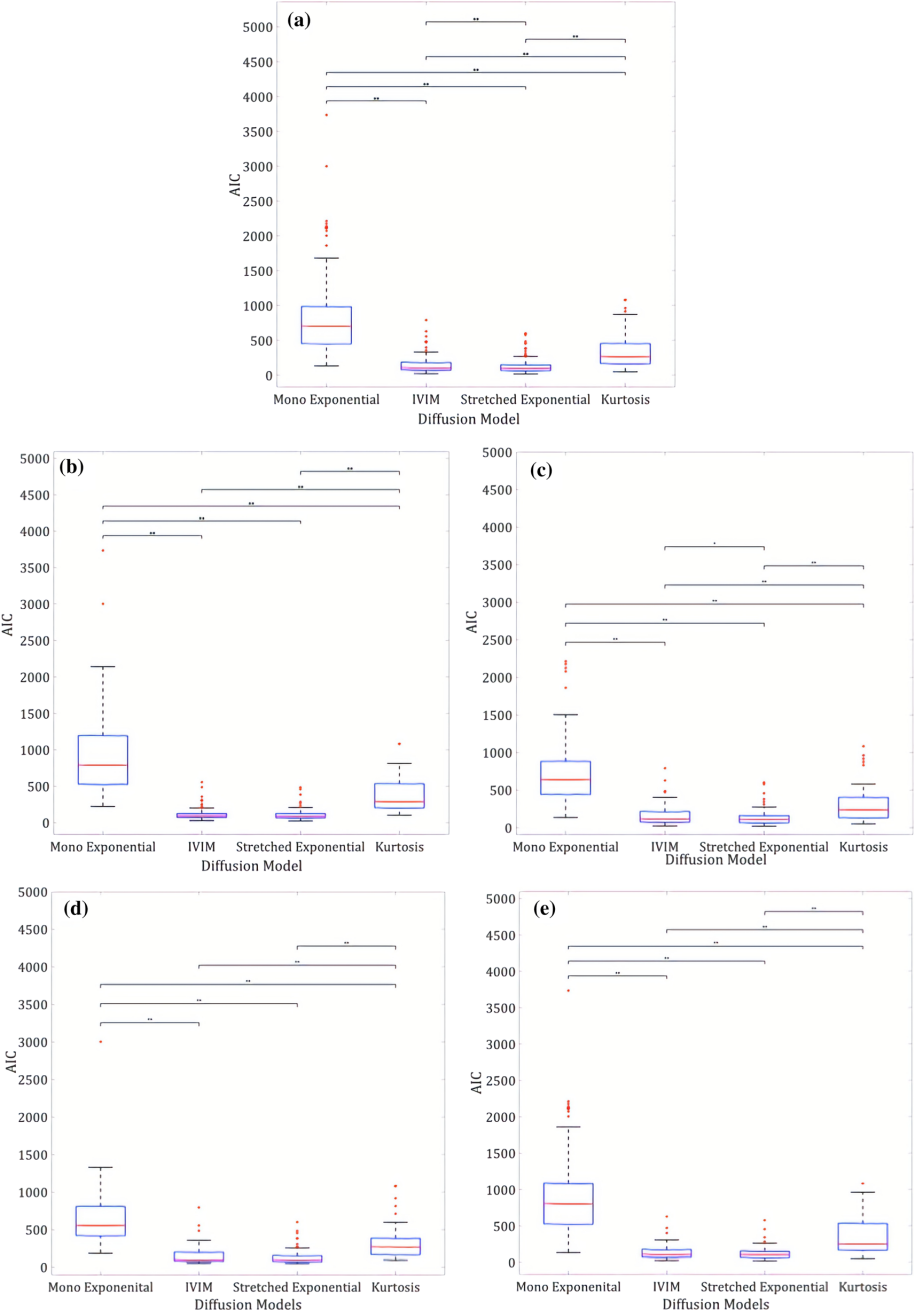
Box and whisker plots highlighting the distribution of AIC (Akaike Information Criterion) values for different diffusion models in Wilms’ tumours. The ends of the blue boxes represent the 25th and 75th percentiles, the red line indicates the median. *significant differences *p* < 0.05, **significant differences *p* < 0.001. **a** Entire Cohort, **b** Pre-chemotherapy, **c** Post-Chemotherapy, **d** 7 b values, **e** 8 b values

**Fig. 4:**
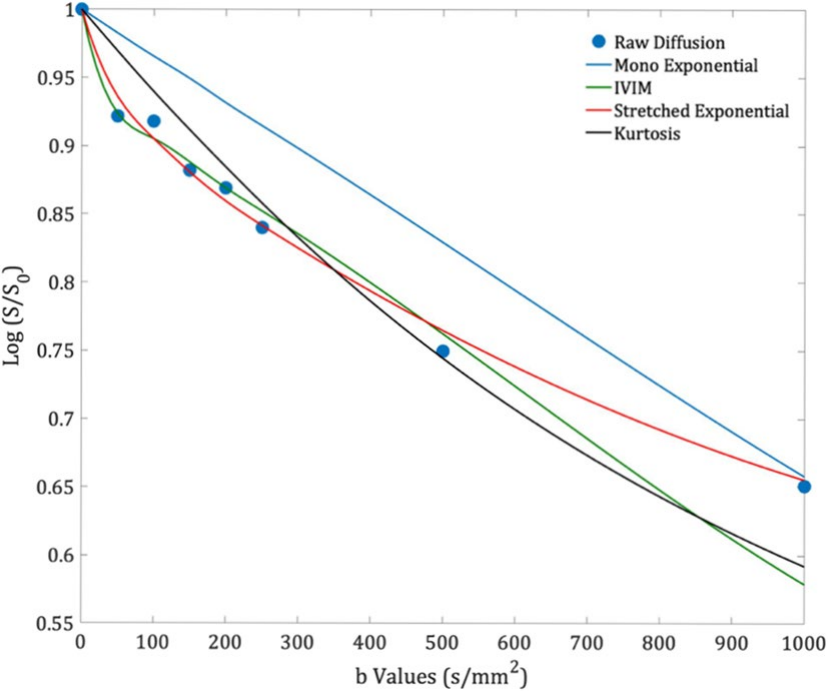
An example of the model fits to the diffusion decay signal in a single voxel (8 b values) of a post-chemotherapy Wilms’ tumour (age at scan: 4.03 years)

Figure 3 shows the boxplots of each condition, with significance bars highlighting the post hoc test results using the Bonferroni correction. In all conditions AIC values for the mono-exponential model were significantly higher than the other three models, indicating that this was the least appropriate model for the Wilms’ tumour data. For the entire Wilms’ tumour and post-chemotherapy cohorts, stretched exponential was the best model for fitting the diffusion data, as this provided the lowest AIC values. Figure 4 shows an example of how well the models fit to the diffusion decay signal in a single voxel of a post-chemotherapy Wilms’ tumour.

For the pre-chemotherapy cohort and when data were split into 7 and 8 b value ranges, both IVIM and stretched exponential were deemed to be the most appropriate models, with no significant difference between the AIC values for these two models.

**Fig. 5:**
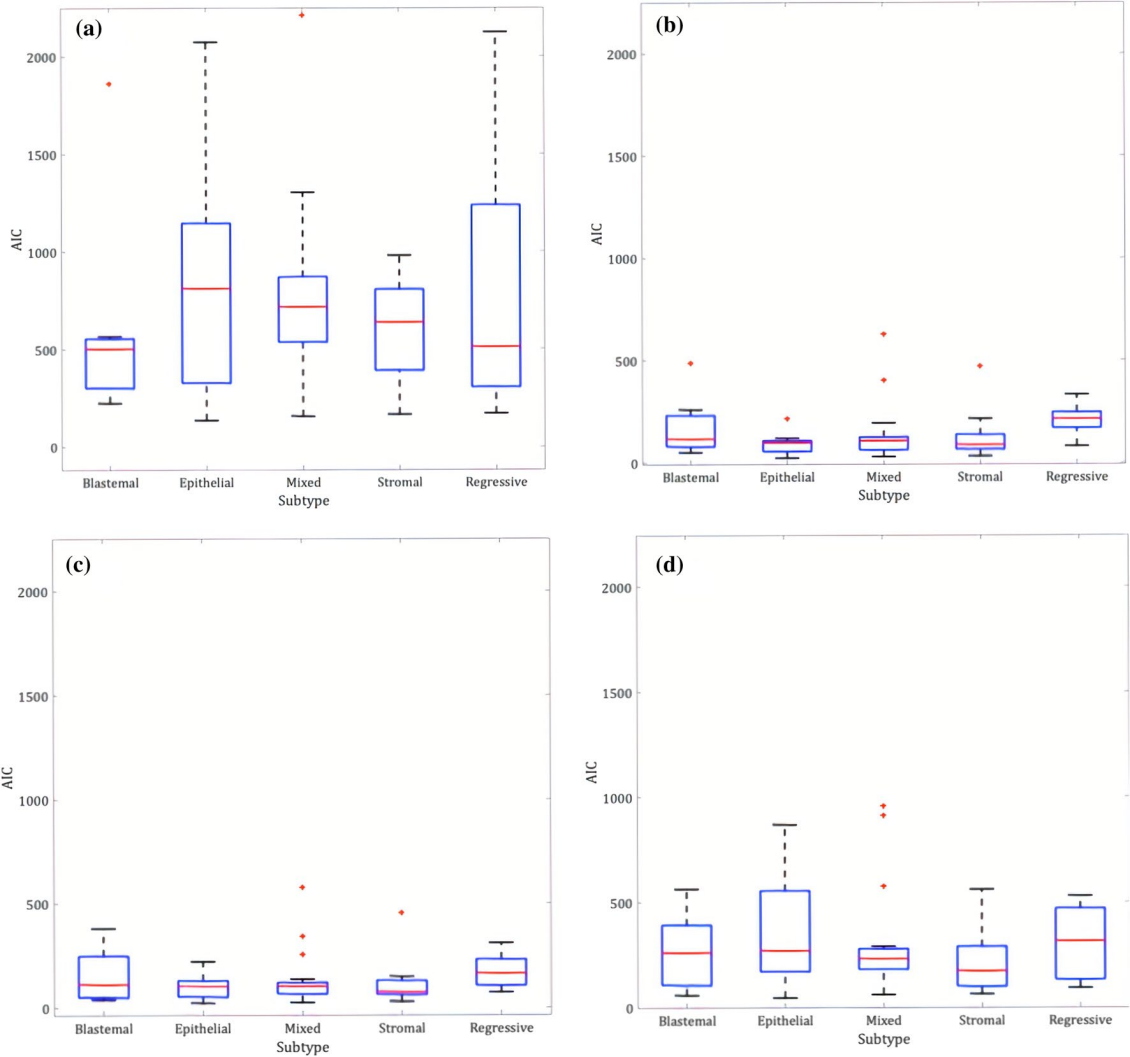
Box and whisker plots highlighting the distribution of AIC (Akaike Information Criterion) values for different subtypes of Wilms’ tumour using various diffusion models. The ends of the blue boxes represent the 25th and 75th percentiles, the red line indicates the median. No significant differences were found (*p* > 0.05) **a** Mono exponential, **b** IVIM, **c** Stretched Exponential, **d** Kurtosis

Additionally, one-way ANOVAs were used to investigate whether the best fit model was related to Wilms’ tumour histological subtypes, using the post-chemotherapy data as they were the closest timepoints to histology. Only one tumour was classified as necrotic and was therefore removed from this section of the analysis. Figure 5 shows the AIC values for each subtype based on different diffusion models. There were no significant differences between AIC values across the subtypes (blastemal [*n* = 7], epithelial [*n* = 9], mixed [*n* = 18], stromal [*n* = 13], regressive [*n* = 8]), for any of the models (*p* > 0.05).

### Normal kidney results

One-way ANOVAs with a Greenhouse–Geisser correction also revealed that AIC values differed significantly between diffusion models, in all conditions for the normal kidney data: entire cohort: (*F*(1.51, 85.2) = 276.07, *p* = 2.57 × 10^–30^), pre-chemotherapy: (*F*(1.43, 41.13) = 119.38, *p* = 1.16 × 10^–14^), post-chemotherapy: (*F*(1.16, 42.81) = 157.33, *p* = 1.10 × 10^–15^, 7 b values: (*F*(1.14, 34.10) = 90.49, *p* = 1.06 × 10^–11^), and 8 b values: (*F*(1.14, 49.10) = 193.30, *p* = 1.19 × 10^–19^).

**Fig. 6:**
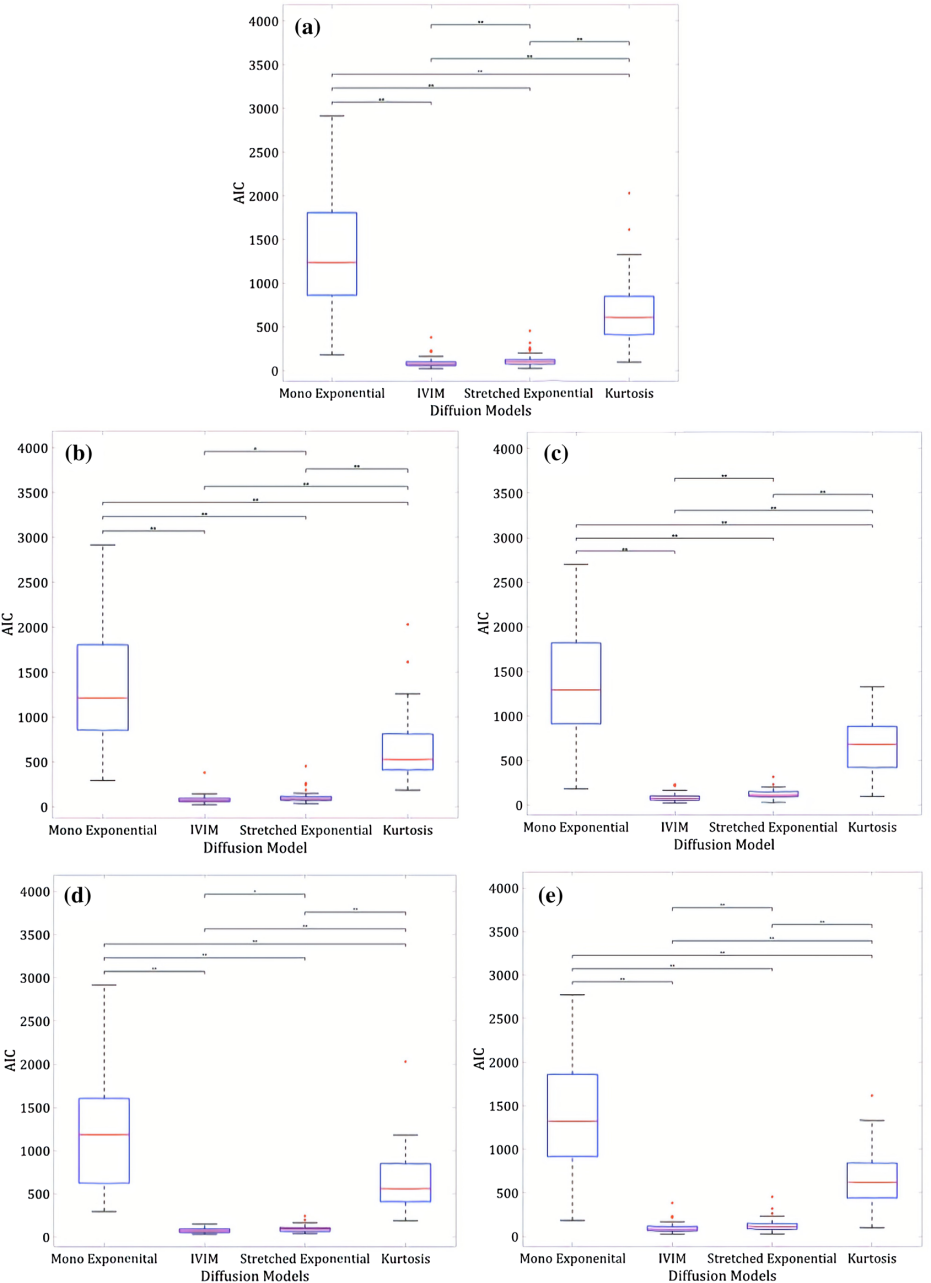
Box and whisker plots highlighting the distribution of AIC (Akaike Information Criterion) values for different diffusion models in normal kidney data. The ends of the blue boxes represent the 25th and 75th percentiles, the red line indicates the median. *significant differences *p* < 0.05, **significant differences *p* < 0.001. **a** Entire Cohort, **b** Pre-chemotherapy, **c** Post-Chemotherapy, **d** 7 b values, **e** 8 b values

**Fig. 7:**
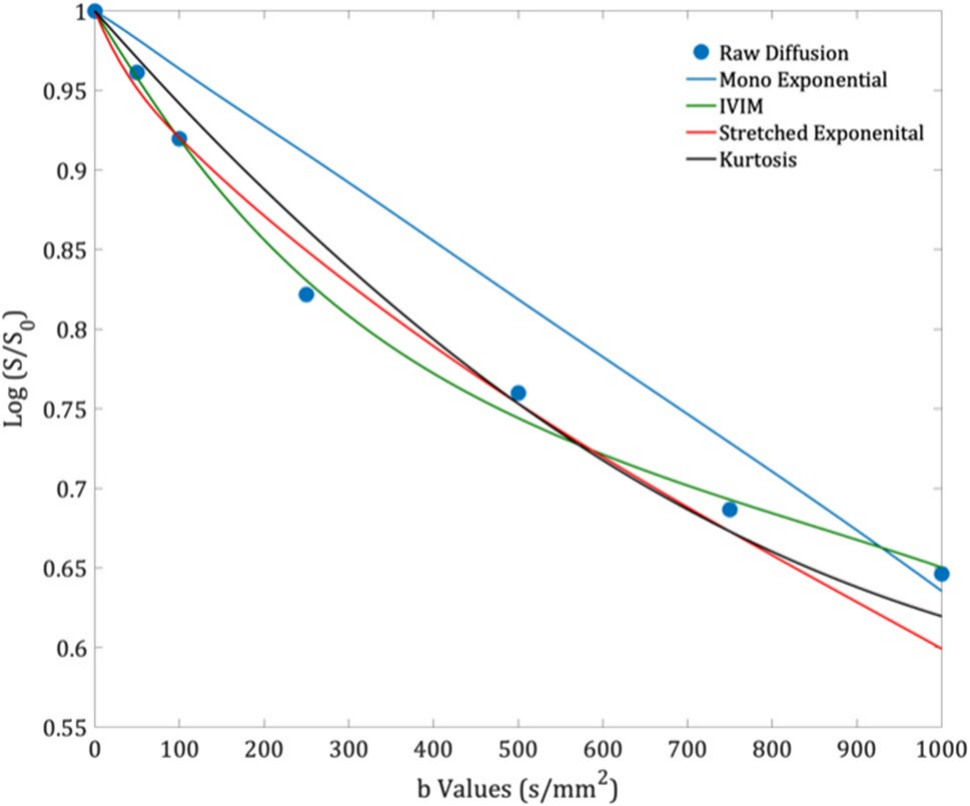
An example of the model fits to the diffusion decay signal in a single voxel (7 b values) in the contralateral normal renal tissue of a post-chemotherapy Wilms’ tumour (age at scan: 2.44 years)

Figure 6 shows the boxplots of each condition, with significance bars highlighting the post hoc test results using the Bonferroni correction. The normal kidney data provided similar results to the Wilms’ tumour data: in all conditions AIC values for the mono-exponential model were significantly higher than the other three models, indicating that this was the least appropriate model for the normal kidney data. Unlike the Wilms’ tumour data, for all conditions, the normal kidney data showed that IVIM provided the lowest AIC values, indicating it was the most appropriate model for this diffusion data. Figure 7 shows an example of how well the models fit to the diffusion decay signal in the contralateral normal kidney data of a post-chemotherapy Wilms’ tumour patient.

## Discussion

This study compared four models of diffusion (mono exponential, IVIM, stretched exponential and kurtosis) based on how well they fit to the DWI signal decay, according to AIC. These comparisons were made in Wilms’ tumours, both pre- and post-chemotherapy, and on the contralateral unaffected kidney, as a measure of normal renal tissue. The diffusion data came from both 7 and 8 b value ranges. For the Wilms’ tumour data, it was shown that the stretched-exponential model provided the best fit overall. This result was maintained when analysis was confined to the post-chemotherapy group. However, when analysis was focused on pre-chemotherapy data and when separated by b value acquisition there were no significant differences between IVIM and stretched exponential, with both models providing the lowest AIC values. Additionally, there were no particular model preferences when the tumours were grouped by histological subtype. For the normal kidney data IVIM provided the best fit in all analyses. The mono-exponential model was shown to be the least appropriate model according to AIC; providing consistently significantly higher AIC values compared to the other models for both the Wilms’ tumour and normal kidney datasets.

The main finding from this investigation was that non-Gaussian models provided better descriptions of the diffusion data compared to mono exponential, in both Wilms’ tumour and normal renal tissue. The deviation from a mono-exponential decay has been previously highlighted and explored: it has been shown that there was a rapid decline in signal at lower b values followed by a more gradual decline at higher b values in the liver [19]. This initial decline was suggested to be due to vascular perfusion, as lower b values are thought to be sensitive to signal attenuation from perfusion [9], making the IVIM model well suited to these data. This has been shown to be the case in healthy renal tissue [20, 21], where signal was shown to be bi-exponential as opposed to mono exponential; as the kidney is a well-perfused organ. The present study supports these findings as IVIM was favoured over the other models in normal renal tissue. This finding was maintained in the post-chemotherapy normal kidney dataset, suggesting that treatment did not affect the normal kidney tissue in a way which could be detected by the DWI data.

The stretched-exponential model provided a good fit to the DWI Wilms’ tumour data. The previously mentioned studies in rectal cancer and healthy rectal tissue [12], prostate cancer bone metastasis [13] and ovarian cancer [14], all showed this model to provide the best fit to DWI data when compared to IVIM and mono exponential. The stretched-exponential model provides two parameters, α and DDC; while the exact physiological basis of α is unknown, it is thought to represent tissue heterogeneity, with a lower value suggesting a more heterogenous environment [10]. As Wilms’ tumour tissue is very heterogenous, it is unsurprising that the stretched-exponential model describes these data well.

In addition to the stretched-exponential model, IVIM provided an equally good fit to the pre-chemotherapy Wilms’ tumours, whereas this was not case post-treatment. Following treatment there is likely to be an increase in necrotic tissue and thus a decrease in perfusion, therefore IVIM (a model which focuses on perfusion effects) may become less suitable.

No particular histological subtype appeared to favour a certain model, however, the numbers in each group were small. Furthermore, it is important to note that histological subtypes are defined after analysing only a subsection of the entire tumour volume. Wilms’ tumours are very heterogeneous and across a single tumour, there will be areas of distinct cellular environments. The voxels within these distinct regions may have shown variable diffusion model preferences. However, due to the lack of advanced histology this analysis was not possible.

Using non-Gaussian models may not only provide better fits to the data but may also provide additional clinical information. For example, it has been shown that D_k_ from kurtosis could provide higher diagnostic accuracy compared to ADC in differentiating tumour from non-tumour in pancreatic cancer [22]. Additionally, α (stretched exponential) had higher levels of sensitivity and specificity when discriminating between minimal fat angiomyolipoma and renal cell carcinoma compared to ADC [23]. Furthermore, both D and f (IVIM) have shown promise in highlighting kidney function, with both parameters being related to estimated glomerular filtration rate in those with chronic kidney disease [24]. Therefore, non-Gaussian models may also have the potential to provide further information about the renal tissue microstructure.

While the mono-exponential model did not provide the best fit to the DWI data, it does not mean that it should not be used clinically. As previously mentioned, ADC has been shown to be clinically useful in Wilms’ tumour [4–8]. Additionally, ADC does not require multiple b values, which is a benefit as many centres may not acquire DWI with multiple b values as standard. Therefore, despite the present study showing a deviation from a mono-exponential signal decay, it is important to be aware that while the model may not be the best descriptor of the DWI data, it is nonetheless clinically useful.

The method for selecting the model which provided the best fit is a potential weakness of this study. AIC takes into account the complexity of the model and goodness of fit, and therefore seemed an appropriate choice for model comparison and selection. It is important to consider if one model is clearly the best for the entire tissue or if there is only a small difference between the models. This was previously highlighted by Manikis et al. [25] in rectal cancer, where although overall mono exponential was preferred to IVIM, there was high heterogeneity across the tissue. This was also demonstrated in Wilms’ tumours in the present study, with both IVIM and stretched-exponential models demonstrating good fits to the data. With this in mind, one should be cautious before claiming that a particular model best fits the data, as it may be that many models are near equal in fitting quality.

Furthermore, the maximum b value of 1000 s/mm^2^ may have been a limitation for the kurtosis model as it becomes more sensitive at higher b values [26]. Therefore, with a more optimised b value range this model may have performed better than with the current data. However, in a study regarding the feasibility of kurtosis in the kidneys a maximum of b = 1000 s/mm^2^ was also used [27]. Additionally, the present study wanted to focus on fitting to routinely acquired clinical data which does not have extremely high b values. This sentiment has also been suggested in previous work which compared the mono-exponential model to kurtosis in the liver also using a maximum of b = 1000 s/mm^2^ [28].

Overall this study demonstrated that the mono-exponential model does not fit DWI data as well as IVIM, stretched exponential or kurtosis in Wilms’ tumour tissue or normal renal tissue. Additionally, there was no model preference for the distinct cellular subtypes. IVIM provided the best fit for the normal renal tissue, and in Wilms’ tumours both IVIM and stretched-exponential models provided the best descriptors of the data. ADC is frequently used in clinical research and therefore the assumption is that the signal decay is mono exponential. However, these results suggest that in Wilms’ tumour and normal renal tissue, the DWI signal does not exhibit a mono-exponential decay. Therefore, utilising other models may provide more accurate representations of the underlying tissue environment, and the derived parameters may provide clinically useful information.
